# Mendelian Randomization Studies of Coffee and Caffeine Consumption

**DOI:** 10.3390/nu10101343

**Published:** 2018-09-20

**Authors:** Marilyn C. Cornelis, Marcus R. Munafo

**Affiliations:** 1 Department of Preventive Medicine, Northwestern University Feinberg School of Medicine, Chicago, IL 60611, USA; 2 MRC Integrative Epidemiology Unit (IEU) at the University of Bristol, UK Centre for Tobacco and Alcohol Studies, School of Psychological Science, University of Bristol, Bristol BS8 1TU, UK; marcus.munafo@bristol.ac.uk

**Keywords:** Mendelian Randomization, coffee, caffeine, behavior, causality, genetic epidemiology, epidemiological methods

## Abstract

Habitual coffee and caffeine consumption has been reported to be associated with numerous health outcomes. This perspective focuses on Mendelian Randomization (MR) approaches for determining whether such associations are causal. Genetic instruments for coffee and caffeine consumption are described, along with key concepts of MR and particular challenges when applying this approach to studies of coffee and caffeine. To date, at least fifteen MR studies have investigated the causal role of coffee or caffeine use on risk of type 2 diabetes, cardiovascular disease, Alzheimer’s disease, Parkinson’s disease, gout, osteoarthritis, cancers, sleep disturbances and other substance use. Most studies provide no consistent support for a causal role of coffee or caffeine on these health outcomes. Common study limitations include low statistical power, potential pleiotropy, and risk of collider bias. As a result, in many cases a causal role cannot confidently be ruled out. Conceptual challenges also arise from the different aspects of coffee and caffeine use captured by current genetic instruments. Nevertheless, with continued genome-wide searches for coffee and caffeine related loci along with advanced statistical methods and MR designs, MR promises to be a valuable approach to understanding the causal impact that coffee and caffeine have in human health.

## 1. Introduction

Coffee is one of the most widely consumed beverages in the world. Consumption patterns vary by country with larger per capita consumptions reported for Nordic countries, such as Finland (12.2 kg), Sweden (10.1 kg) and Norway (8.7 kg) compared to other countries such Brazil (5.9 kg), Netherlands (5.3 kg), USA (4.5 kg), Australia (4.0 kg), Russia (1.7 kg), China (0.8 kg) and Turkey (0.7 kg) [1]. For most populations, regular coffee is the primary dietary source of caffeine; a psychostimulant also present in tea, cola, and cocoa products. Absorption and exposure to caffeine from these different sources is similar although a slight delay in absorption has been reported for cola and chocolate [2,3,4]. Roasted coffee also contains unique polyphenols (i.e., chlorogenic acid) and melanoidins that are major contributors to antioxidants in diet [5,6]. Boiled or unfiltered coffee contains diterpenoids, including cafestol and kahweol [7]. Trigonelline, magnesium, potassium, niacin, lignans, as well as heterocyclic amines and acrylamide have also been detected in the beverage [8,9,10,11,12]. With widespread popularity and availability of coffee, there is increasing public and scientific interest in the potential health consequences of its regular consumption. Traditional epidemiology has been fundamental to our increased knowledge on habitual coffee intake and health; but while a highly efficient and relevant approach, it has several limitations that warrant consideration when interpreting the results [13]. Among these is establishing causal associations. The current perspective focuses on Mendelian Randomization (MR) approaches for determining a causal role of habitual coffee and caffeine intake on health. Because coffee and dietary caffeine intake are highly correlated we focus on both exposures. We first provide a brief review of coffee, caffeine and health. We follow with key concepts of the MR approach and particular challenges when applying it to studies of coffee and caffeine. Recent MR studies of coffee, caffeine and health are discussed, and we conclude with future directions for the field.

## 2. Coffee, Dietary Caffeine and Health

A recent umbrella review considered data from 201 meta-analysis of epidemiological studies of 67 unique health outcomes, and concluded that coffee likely has a beneficial role in reducing risk of type 2 diabetes (T2D), cardiovascular diseases (CVD), several cancers and Parkinson’s disease (PD), but that high caffeine intake is likely harmful on pregnancy outcomes, such as low birth weight and pregnancy loss [14]. Overall, coffee consumption seems generally safe within usual levels of intake (i.e., at 3 to 4 cups a day) and more likely to benefit health than harm [14]. Rigorous reviews of caffeine toxicity conclude that consumption of up to 400 mg caffeine/day (equivalent to ~4 cups of coffee) in healthy adults, or 2.5 mg/kg/day for children and adolescents is not associated with overt adverse effects [15] and thus generally support the overall findings on habitual coffee intake and health [14]. Meanwhile, the Diagnostic and Statistical Manual of Mental Disorders (DSM-V) lists caffeine intoxication and withdrawal as disorders, and have added ‘caffeine use disorder’ to ‘Conditions for Further Study’ [16]. Much of our knowledge pertaining to habitual coffee and caffeine intake on risk of chronic disease has been limited to observational research [14,15]. Inferring causality from observational data is difficult, due to potential residual confounding and reverse causality [17]. For example, in some populations coffee consumption is highly correlated with disease risk factors, such as smoking. Participants might acknowledge their true coffee behavior, but underreport their smoking behavior. As a consequence the coffee intake variable will continue to convey information about smoking even after adjustment for measures of smoking [18]. Coffee drinkers may also have reduced their coffee intake in light of disease symptoms or diagnosis, which might result in an apparent, but non-causal protective association between coffee and the disease [19]. Observational studies also provide no insight to mechanisms linking coffee to health. Coffee contains caffeine, but also hundreds of other chemicals that might benefit or harm health via different biological pathways [9]. Randomized trials of coffee consumption and disease outcomes would require long-term adherence to high or no coffee consumption, which is challenging given strong coffee consumption habits [20].

## 3. Mendelian Randomization (MR)

MR is a method of using the association of variation in genes with biomarkers or modifiable exposures to examine the causal effect of these biomarkers and exposures on disease outcomes in observational studies. The underlying principle of MR is that if a genetic variant alters the level of an exposure of interest, then this genetic variant should also be associated with disease risk and to the extent predicted by the effect of the genetic variant on the exposure [21,22]. According to Mendel’s Law of Inheritance, alleles segregate randomly from parents to offspring. Thus, offspring genotypes are unlikely to be associated with confounders in the population. Moreover, germ-line genotypes are fixed at conception and so precede the observed variables, avoiding issues of reverse causation [23]. MR studies are often described as natural RCTs, but there are important differences [24]. For example, RCTs are usually of short duration while an individual’s genetics generally reflect life-long exposures [21,24,25].

MR relies on a number of assumptions, in particular that the genetic variants(s): (1) Is associated with the modifiable exposure of interest, (2) is not associated with confounders of the exposure to outcome association and (3) only influences the outcome through the exposure of interest [17]. The first assumption is the only one that can be formally tested, but MR methods and study designs have advanced much over the last few years and now include methods that are robust to potential violations of assumptions (2) and (3). It is increasingly widely used as a causal inference method in epidemiology. One-sample (genetics, exposure and outcome measured in the same sample) and two-sample (exposure and outcome measured in different samples) are the most common MR study designs. The latter is advantageous in situations where it is difficult to measure exposure and outcome in the same sample and can also be performed on publicly available genome-wide association study (GWAS) data (summary-level data). When possible, an instrument (genetic marker of exposure) that combines the effects of many SNPs is used to boost power while also addressing MR assumption violations (see below). The basic method for summary-level data, inverse-variance weighted (IVW), uses a fixed effects meta-analysis approach to combine the Wald ratio estimates of the causal effect (SNP-outcome effect divided by the SNP-exposure effect [26]) obtained from different SNPs, but assumes all SNPs are valid instruments or are invalid in such a way that the overall bias is zero [27,28]. The IVW is generally equivalent to the two-stage least squares estimate commonly used with individual level data.

## 4. Genetic Determinants of Coffee and Caffeine Consumption

Opportunities for MR studies of coffee and health have been made possible by the success of GWAS, which have identified multiple genetic variants associated with self-reported habitual coffee and caffeine consumption (Table 1) [29,30,31,32,33]. Loci near *ADORA2A*, *BDNF* and *SLC6A4* likely act directly on coffee drinking behavior by modulating the acute psychostimulant and rewarding properties of caffeine; driving factors for coffee drinking and caffeine use [34]. However, loci near *AHR*, *CYP1A2*, *POR*, and *ABCG2* generally present with the largest effect sizes and likely impact drinking behavior indirectly by altering the metabolism of caffeine and thus the physiological levels of this compound available for its psychostimulant effects. Only one locus is implicated in the sensory properties of coffee (*OR8U8*). Others have no obvious role in coffee or caffeine consumption, but have previously been associated with other traits in GWAS notably obesity, glucose and lipids [35,36,37,38]. GWAS and smaller follow-up studies have linked these loci to consumption of regular coffee, decaffeinated coffee, tea, total caffeine and water [31,39,40]. A subsequent GWAS of circulating caffeine metabolite levels further informed the roles of these loci in coffee and caffeine consumption behavior, but also identified variants near *CYP2A6* associated with paraxanthine-to-caffeine ratio (index for caffeine metabolism), that were nominally associated with drinking behavior [41]. Importantly, genetic variants leading to increased coffee/caffeine consumption associate with lower circulating caffeine levels and higher paraxanthine-to-caffeine ratio suggesting a fast caffeine metabolism phenotype. Thus, many of the loci affecting coffee and caffeine drinking behavior do so by modulating the physiological levels of caffeine.

## 5. Key Challenges to MR Studies of Coffee and Caffeine

Despite progress in the identification of robust genetic variants for coffee and caffeine consumption, efforts to apply these variants to MR studies of coffee and caffeine have been met with challenges, such as trait heterogeneity, pleiotropy and collider bias as discussed below. Limitations in the conduct and interpretation of MR studies more generally, along with potential solutions, have been reviewed in detail elsewhere [23,25,42], and include weak instrument bias, lack of reliable genetic instruments, population stratification, low statistical power (and therefore wide confidence intervals around causal estimates), linkage disequilibrium (LD) and the Winner’s Curse phenomenon (i.e., the tendency for effect sizes in initial studies to be inflated).

### 5.1. Trait Heterogeneity

The most comprehensive (and therefore powerful) genetic instrument employed in an MR study of coffee will reflect multiple aspects of coffee drinking behavior (Table 2), such as caffeine metabolism, reward-response and potentially taste. Such heterogeneity does not preclude causal inference, but it does limit the ability to infer causality for particular dimensions of coffee (e.g., caffeine vs non-caffeine) and makes interpretation of MR analyses more difficult [23,25]. An instrumental variable (IV) that narrows in on a particular aspect of coffee drinking might also face issues of interpretation. For example, genetically-inferred ‘fast’ and ‘slow’ caffeine metabolizers may consume different amounts of the same type of coffee, but their circulating caffeine levels may not be different. However, circulating levels of non-caffeine constituents of coffee *will* differ. Alternatively, given the same amount and type of coffee consumed, slow caffeine metabolizers will, on average, have higher circulating caffeine levels than fast caffeine metabolizers. Circulating levels of non-caffeine constituents will generally be the same. Because most of the SNPs associate with caffeine intake, and not exclusively coffee intake, the genetic instrument for coffee might also reflect exposure to other dietary sources of caffeine, which might confound or mask any causal relationship between coffee and outcome [43]. Although MR studies are thought to be relatively protected against exposure measurement error, this is less likely to be the case for an MR study of coffee or caffeine [20]. For example, the genetic predisposition to drink coffee, due to an increased caffeine metabolism might also impact preference for regular strong coffee over other coffee types. Taken together, it is important to specify the hypothesis being tested a priori, select the optimal IV and sample for analysis, and consider alternate explanations for positive or null results.

### 5.2. Pleiotropy

Pleiotropy can violate MR assumption 3, which requires that the genetic variant only influences the outcome through the exposure of interest. Vertical pleiotropy does not violate MR assumption 3 and occurs when the genetic variant is associated with a factor on the pathway between the exposure and outcome, but only because of its effect on the exposure [58]. Horizontal (or biological) pleiotropy occurs when a genetic variant is associated with multiple exposures or traits and is therefore a violation of MR assumption 3 [17,58]. Seven of the fourteen loci associated with coffee or caffeine consumption are also associated with other traits based on GWAS [35] (Table 1). Whether this results from horizontal pleiotropy or a true causal relationship between coffee and these other traits is unclear. Nevertheless, since it is not possible to prove assumption 3 holds for all SNPs in an MR study its becoming common practice to implement extensions of the basic MR methodology that detect the presence of pleiotropy and account for it in causal estimates of the exposure [59]. Random effects IVW or weighted generalized linear regressions are simple options [22,60,61], but common methods that explicitly account for pleiotropy include MR-Egger regression [62], and the weighted-median estimate [63]. Newer methods include MR-PRESSO [64] and generalized summary MR (GSMR) [65]. Each approach relies on different (and largely uncorrelated) assumptions, and therefore the use of multiple approaches allows triangulation; if all provide consistent causal estimates we can be more confident that a true causal effect exists.

### 5.3. Collider Bias

When individual-level data are available, a common strategy is to restrict SNP-outcome analysis to coffee drinkers arguing that the SNPs are associated with coffee drinking (heaviness) and thus causal relationships should only be observed among coffee drinkers (a form of gene-environment interaction) [43,44,48,49,56,59,66]. SNP-outcome associations among non-drinkers (‘negative control sample’) would suggest a violation in at least one of the assumptions [59,66]. However, this strategy introduces potential for collider bias given that several loci associated with coffee intake also distinguish between non-drinker and heavy coffee drinkers [31]. Collider bias occurs when the exposure and outcome of interest independently influence a third risk factor, and this third risk factor is conditioned upon, either through statistical adjustment or stratification [67,68,69]. This bias will also apply to the genetic correlates of the exposure and outcome. Indeed, MR studies of coffee intake among the Copenhagen population provided evidence for collider bias [43,44]. For example, among coffee-abstainers, the genetic IV for coffee intake was inversely associated with age. Since age was a risk factor for the outcome and was strongly associated with coffee intake, but among coffee consumers only, the IV-age association in the ‘negative control sample’ likely arises from collider bias [43].

## 6. MR Studies of Coffee, Caffeine and Health

Table 2 summarizes all MR studies of coffee or caffeine and health outcomes published to-date. Studies are in descending order by date of publication (column 1). For each study we extracted the outcome of interest (column 2), the genetic variants used as the IV (column 3), the basic design and approach (column 4), main results (column 5), interpretation or overarching conclusion of the study (column 6) and limitations as acknowledged by study authors (column 7). With one exception [57], all study IVs included at least SNPs near *CYP1A2* and *AHR*—the strongest and most robust variants linked to coffee drinking behavior and caffeine metabolite levels (Table 1). Primary analysis was conducted using predominately regression analyses or IVW meta-analysis for multi-SNP analysis. These were generally followed by weighted median estimates and MR-Egger regressions to address potential assumption violations. In most studies, the exposure of interest was simply defined as coffee consumption or caffeine use. Data from the GWAS of coffee consumption among 91,462 coffee drinkers in the Coffee and Caffeine Genetics Consortium (CCGC) [31] were used in all summary-level data analysis.

Epidemiological studies report a consistent inverse linear association between coffee consumption and T2D [14], which extends to decaffeinated coffee. This is typically interpreted as evidence for non-caffeine constituents of coffee underlying the coffee-T2D relationship [14]. Two studies, using individual-level and summary-level data for up to ~170,000 participants (26,000 T2D cases) provided no evidence in support of a causal association between coffee intake and T2D risk [44,45], which also extended to measures of adiposity, blood pressure, lipid and glucose metabolism [44,45]. Nordestgaard and colleagues [44] additionally examined a BMI IV (SNPs in/near *FTO*, *MC4R* and *TMEM18*) to examine potential reverse causation from BMI to coffee intake, and as a positive control for risk of T2D. The coffee-intake IV was not linked to BMI, but the BMI-IV was positively associated with coffee intake. Interestingly, SNPs included in the BMI-IV were recently shown to associate with coffee consumption in GWAS (Table 1) [33] and so possibly relate to reward mechanisms (the causal pathway) relevant to coffee drinking behavior and obesity and not adiposity per se [33].

Epidemiological studies also suggest coffee intake may reduce risk of CVD, CVD-mortality and all-cause mortality, but with greatest risk reduction with 3 to 5 cups/day (i.e., a non-linear association) [14]. Nordestgaard and Nordestgaard [43] examined all three of these outcomes in 112,509 Danes and observed a similar pattern of benefits associated with coffee consumption over a 6 year follow-up, but no evidence for causality. In the subgroup of coffee drinkers they noted strong positive and plausible LDL-SNP and HRT-SNP associations, but could not rule-out that such associations could have resulted from collider bias [43].

Caffeine, nicotine, alcohol, and cannabis use are highly correlated behaviors [70]. Potential mechanisms include shared genetic and/or shared environmental factors (i.e., common liability) or a causal influence of one on the other [71]. The co-occurrence of coffee/caffeine use with other substance use behaviors has been investigated in four MR studies [46,48,49,51]. Three of these studies employed bidirectional MR [46,49,51], in which IVs for each substance use were used to evaluate causal effects and their direction [23,72]. The first study focused on the association between smoking and caffeine using three approaches: Bivariate genetic modelling in a twin sample, LD score regression with summary level-data and bidirectional MR analysis using individual-levels data [46]. The results suggested shared genetic factors for caffeine/coffee intake and smoking behavior, rather than a causal influence of one behavior on the other. Ware and colleagues [48] specifically focused on the causal role of coffee consumption on smoking heaviness. Two-sample MR analyses indicated that heavier coffee consumption might lead to *reduced* heaviness of smoking. However, their in vitro experiments, and attempt to replicate in the UK Biobank sample of smokers who drank coffee, did not support these initial causal findings, and overall were not consistent with the direction of association reported in observational analysis. Bjorngaard and colleagues [49] also examined coffee and tea drinkers from three population studies using bidirectional MR and provided evidence for a causal relationship of smoking heaviness on coffee and tea intake, but not vice versa. Finally, Verweij and colleagues [51] examined causal relationships among caffeine, smoking, as well as alcohol, and cannabis use with a variation of bidirectional MR that used ‘polygenic scores’. The latter relaxes the significance threshold for GWAS to produce a stronger instrument, but also runs the risk of vertical pleiotropy [59]. Their findings did not support the hypothesis that causal relationships explain the co-occurrence of use of different substances, but are consistent with a common liability model [51].

Alzheimer’s Disease (AD) was investigated by Kwok and colleagues [45], and Larsson and colleagues [50], using the same summary-level data, but employed different multi-SNP IVs. Larsson and colleagues [50] used an IV with SNPs for *AHR*, *CYP1A2*, *MLXIPL*, *POR* and *EFCAB* and reported a suggestive causal relationship between coffee and AD risk, but in the opposite direction to that expected based on observational data. Kwok and colleagues [45], whom did not include the *MLXIPL* SNP in their IV, reported no evidence for a causal relationship. A causal relationship between coffee and cognitive function was also not supported by a separate MR [56]. The latter accounted for the potential non-linear association between coffee and cognitive function by conducting analysis by different levels of coffee intake. An association among non-coffee consumers served as a negative control sample. While collider bias was not acknowledged as a limitation, they noted caution when interpreting their results as the instruments indexing greater caffeine consumption may reflect a faster rate of caffeine clearance, and hence a lower (rather than higher) circulating level of bioactive caffeine [56].

Although data are limited, coffee intake has been linked to lower risk of gout [14]. Larsson and colleagues [53] examined the causal association between coffee and gout, as well as uric acid, a related biomarker. The five SNP-IV (excluding the *ABCG2* SNP, which associates with uric acid) was inversely related to both gout risk and uric acid levels, supporting a causal relationship between coffee drinking and gout.

MR studies have failed to support a causal association between coffee/caffeine intake and epithelial ovarian cancer [52], prostate cancer [47], sleep behaviors [54] and Parkinson’s disease (PD) [55]. The latter finding is in marked contrast to consistent observational and animal experimental data suggesting coffee and caffeine are protective for PD, but rather align with RCTs and suggest “caffeine may neither prevent PD occurring nor be of benefit in those with the condition” [55]. The authors nevertheless noted that potentially causal effects of coffee may not occur exclusively through caffeine [55], suggesting their IV aimed to capture caffeine exposure rather than coffee drinking per se. The most recent coffee MR was applied to osteoarthritis [57] and supported a causal positive relationship between coffee and this outcome. However, the selection of SNPs for the study was unclear and no human observational study has examined coffee and osteoarthritis, so that the findings are largely hypothesis-generating.

Taken together, at least fifteen studies to date have investigated the causal role of coffee or caffeine use in T2D, CVD, AD and cognition, PD, gout, osteoarthritis, cancers, sleep and other substance use behaviors. Single studies investigated and provided support for a causal role of coffee in reducing risk of gout [14] and increasing risk of osteoarthritis [57]. Four studies examined the co-occurrence of caffeine use and other substances with conflicting results [46,48,49,51]. For the remaining outcomes, studies did not provide clear support for a causal role of coffee or caffeine, but often acknowledged limitations (such as low statistical power, pleiotropy and collider bias), such that a causal role cannot yet be ruled out.

## 7. Future Directions

There is continued enthusiasm for understanding the causal role of coffee and caffeine in health. Thus far, most outcomes of interest have been investigated by single studies and thus the significant and null findings warrant confirmation in independent studies. Many outcomes, for which coffee and caffeine have been implicated, have yet to be investigated [14]. Methodological challenges, such as insufficient power, pleiotropy and collider bias are commonly acknowledged. However, conceptual challenges arising from the different aspects of coffee/caffeine use captured by genetic instruments warrant careful consideration going forward. With continued investment in GWAS it may be possible to parse variants related to non-caffeine aspects of coffee from those related to caffeine providing opportunities to identify the causal elements of coffee per se, rather than coffee drinking behavior. The increasing availability of large individual-level data sets and advanced statistical methods means that more sophisticated MR designs might also be considered. For example, the use of polygenic scores might be optimized using the MR robust adjusted profile score (MR-RAPS) method, which weights each variant differently based on effect size and precision of the SNP-exposure association [62]. Given the co-occurrence of coffee drinking and smoking, a factorial MR may be an attractive approach to study the combined causal effects (i.e., interaction) of these behaviors on disease [22]. Individuals can be allocated into either a high or low-SNP score for coffee and then each group further allocated into either a high or low-SNP score for smoking. The causal estimates for each of the resulting four groups on disease could then be determined. A two-step MR may also be used to assess whether an intermediate trait, say a biomarker or metabolite, acts as a causal mediator between coffee drinking and an outcome [73,74]. An IV for coffee drinking is first used to estimate the causal effect of coffee drinking on the potential mediator (step 1). IVs for the potential mediator are then used to assess the causal effect of the mediator on the outcome (step 2). Evidence of association in both steps implies some degree of mediation of the association between coffee drinking and the outcome by the intermediate variable. Finally, multivariable MRs allow multiple exposures to be examined simultaneously, and provide an effect estimate of one conditional on the other (e.g., effects of coffee consumption conditional on circulating caffeine levels) [75]. These alternate MR designs will still require careful attention to challenges and limitations discussed above.

Multiple statistical methods to accommodate different MR violations combined with replication studies and other mechanistic studies will be necessary to support stronger causal relationship between coffee or caffeine intake and health [59]. GWAS of more refined coffee drinking behaviors, and circulating metabolite markers of coffee intake will also be important, but the collection of such data on a large scale will be needed first. Nevertheless, in light of the rapid pace, in which advancements are being made in these areas, MR promises to be an increasingly valuable approach to understanding the causal impact that coffee and caffeine have in human health. 

## Figures and Tables

**Table 1:** Genetic determinants of coffee and caffeine consumption [29,30,31,32,33].

Locus (Index SNP, Coffee/Caffeine Increasing Allele)	Closest Gene(s)	Encoded Protein(s): Function [UniProtKb]	Assoc. with Caffeine Metabolites *	Assoc. with Other Traits †	Hypothesized Link to Caffeine or Coffee Consumption
1q25.2 (rs574367, T)	*SEC16B*	SEC16 Homolog B, Endoplasmic Reticulum Export Factor: Required for secretory cargo traffic from the endoplasmic reticulum to the Golgi apparatus and for normal transitional endoplasmic reticulum organization.	*p* > 0.05	Y	None
2p25.3 (rs10865548, G)	*TMEM18*	Transmembrane Protein 18: Transcription repressor. Sequence-specific ssDNA and dsDNA binding protein, with preference for GCT end CTG repeats. Cell migration modulator, which enhances the glioma-specific migration ability of neural stem cells and neural precursor cells.	*p* > 0.05	Y	None
2p23.3 (rs1260326,C)	*GCKR*	Glucokinase regulatory protein (GKRP): Inhibits glucokinase by forming an inactive complex with this enzyme.	↓↓ *p* < 1 × 10^−5^	Y	Response to caffeine/coffee: May function in the glucose-sensing process of the brain that may influence central pathways responding to caffeine/coffee. Metabolism of caffeine: Inferred by association with caffeine metabolites
4q22 (rs1481012, A)	*ABCG2*	ATP-binding cassette sub-family G member 2: High-capacity urate exporter. Plays a role in porphyrin homeostasis and cellular export of hemin and heme. May play an important role in the exclusion of xenobiotics from the brain. Implicated in the efflux of numerous drugs and xenobiotics.	↑ *p* < 0.05	Y	Metabolism of caffeine: Caffeine/metabolite efflux transporter.
7p21 (rs4410790 C, rs6968554, G)	*AHR*	Aryl hydrocarbon receptor: Ligand-activated transcriptional activator. Activates the expression of multiple phase I and II xenobiotic metabolizing enzymes. Involved in cell-cycle regulation and likely plays a role in the development/maturation of many tissues.	↓↓ *p* < 5 × 10^−8^	N	Metabolism of caffeine: Regulates *CYP1A2* expression.
7q11.23 (rs7800944, C)	*MLXIPL*	Carbohydrate-responsive element-binding protein: Transcriptional repressor.		Y	Response to caffeine/coffee: May regulate transcription of genes (e.g., *GCKR*) implicated in the response to caffeine.
7q11.23 (rs17685, A)	*POR*	NADPH-cytochrome P450 reductase: Required for electron transfer from NADP to cytochrome P450 in microsomes and can also facilitate electron transfer to heme oxygenase and cytochrome B5.	↓ *p* < 0.05	N	Metabolism of caffeine: Required for CYP1A2 catalytic activity.
11p13 (rs6265, C)	*BDNF*	Brain-derived neurotrophin factor: During development, promotes survival and differentiation of selected neuronal populations of the PNS and CNS. Major regulator of synaptic transmission and plasticity at adult synapses in many regions of the CNS.	*p* > 0.05	Y	Response to caffeine: Modulates neurotransmitters potentially mediating the rewarding response to caffeine.
11q12.1 (rs597045, A)	*OR8U8*	Olfactory Receptor Family 8 Subfamily U Member 8: Odorant receptor	*p* > 0.05	N	Smell/taste perception of coffee
14q12 (rs1956218, G)	*AKAP6*	A-Kinase Anchoring Protein 6: Binds to type II regulatory subunits of protein kinase A and anchors/targets them to the nuclear membrane or sarcoplasmic reticulum. May act as an adapter for assembling multiprotein complexes.	*p* > 0.05	N	None
15q24 (rs2470893 T, rs2472297, T)	*CYP1A1, CYP1A2*	Cytochrome P450 1A1/2: Cytochromes P450 are a group of enzymes involved in NADPH-dependent electron transport pathways. They oxidize a variety of compounds, including steroids, fatty acids, and xenobiotics.	↓↓ *p* < 5 × 10^−8^	N	Metabolism of caffeine: CYP1A2 metabolizes >95% of caffeine.
17q11.2 (rs9902453, G)	*EFCAB5* *SLC6A4*	EF-hand calcium-binding domain-containing protein 5: Unknown Sodium-dependent serotonin transporter: In CNS, regulates serotonergic signaling via transport of serotonin molecules from the synaptic cleft back into the presynaptic terminal for reuse.	*p* > 0.05	N	Response to caffeine/coffee: Serotonin may mediate the rewarding response to caffeine.
18q21.32 (rs66723169, A)	*MC4R*	Melanocortin 4 Receptor: Receptor specific to the heptapeptide core common to adrenocorticotropic hormone and alpha-, beta-, and gamma-MSH. Plays a central role in energy homeostasis and somatic growth.	*p* > 0.05	Y	None
22q11.23 (rs2330783, G)	*SPECC1L-ADORA2A*	Adenosine A2a Receptor: Receptor for adenosine. The activity of this receptor is mediated by G proteins, which activate adenylyl cyclase.	↑ *p* < 0.05	N	Response to caffeine/coffee: Caffeine blocks this receptor, which mediates some of the psychostimulant effects of caffeine.

* SNP is associated with (i) higher blood levels of caffeine (↑); (ii) lower blood levels of caffeine (↓); or (iii) lower blood levels of caffeine and higher paraxanthine-to-caffeine ratio (↓↓). † GWAS (genome-wide association study) catalogue traits unrelated to caffeine or coffee. Y, Yes; N, No.

**Table 2:** Mendelian Randomization (MR) studies of coffee and caffeine consumption.

Study	Outcome	Instrumental Variable (IV)	Design & Approach	Results	Interpretation	Limitations Reported
Nordestgaard et al. 2015 [44]	Obesity, metabolic syndrome, T2D and related measures (BMI, WC, height, weight, SBP, DBP, TGs, TC, HDL, glucose)	5-SNPs *AHR, CYP1A2* Score and single SNPs	One-sample Individual-level data 2SLS *n* ≤ 93,179 Copenhagen General Population Study (CGPS) and the Copenhagen City Heart Study (CCHS). Summary-level data Wald ratio, IVW T2D only DIAGRAM (*n* ≤ 78,021)	Observational: Coffee significantly reduced risk of obesity, metabolic syndrome and T2D Coffee significantly increased BMI, WC, weight, height, SBP, DBP, TGs, and TC and decreased HDL SNP-outcome: NS Similar results when individuals were stratified into coffee drinkers and coffee abstainers however, among those without coffee intake, blood pressure was lower with higher coffee-intake allele score	No evidence supporting a causal relationship between coffee and outcomes	Underpowered IV Pleiotropy Collider Bias
Nordestgaard & Nordestgaard, 2016 [43]	CVD (IHD, IS, IVD) All-cause and CVD mortality	2-SNPs *AHR, CYP1A2* Score and single SNPs	One-sample Individual-level data 2SLS *n* ≤ 112,509 CGPS, CCHS and Copenhagen Ischaemic Heart Disease Study (CIHDS) 3822 IHD cases 1708 IS cases 4971 IVD cases 971 CVD deaths 5422 total deaths Summary-level data Wald ratio, IVW IHD only Cardiogram (*n* = 80,517) and C4D (*n* = 30,433)	Observational: U-shaped association between coffee intake and IHD, IS, IVD and all-cause mortality. Lowest risk with medium coffee intake compared with no coffee intake. SNP-outcome: NS Similar results when individuals were stratified into coffee abstainers, coffee drinkers, coffee drinkers excluding tea and cola drinkers.	No evidence supporting a causal relationship between coffee and outcomes	Underpowered IV Pleiotropy Collider Bias (stratified analysis) Confounding by other caffeine containing-beverages Cannot rule out non-linear effects of coffee on outcomes
Kwok et al., 2016 [45]	T2D, IHD, depression, Alzheimer’s disease, lipids, glycemic traits, adiposity or adiponectin	9-SNPs *AHR, CYP1A2*(2), *GCKR, MLXIPL, POR, EFCAB5, BDNF, ABCG2* 5 SNPs *AHR, CYP1A2*(2), *POR, EFCAB5* 3 SNPs *AHR, CYP1A2*(2)	Two-sample Summary-level data Multiple published GWAS WME	9 SNPs: ↑T2D, ↓TGs, ↑BMI, ↑WHR, ↑IR 5 SNPs: NS 3 SNPs: NS	No evidence supporting a causal relationship between coffee and outcomes	Confounding (Population stratification) Pleiotropy Cannot rule out non-linear effects of coffee on outcomes
Treur et al., 2016 [46]	Smoking behavior Coffee intake Caffeine use	1-SNP for smoking heaviness (*CHRNA3*) 8-SNP score for coffee intake *AHR, CYP1A2, GCKR, MLXIPL, POR, EFCAB5, BDNF, ABCG2*	Individual-level data Bivariate genetic modelling (SEM) *n* = 10,368 current smoking (y/n) caffeine use (high/low) coffee use (high/low) Bidirectional MR Regression analyses *n* = 12,319 Self-reported caffeine use (mg/day), coffee use (cups/day), cigs/day, smoking initiation and persistence Summary-level data LD score regression CCGC Tobacco, Alcohol and Genetics Consortium (TAG): cigs/day, smoking initiation and persistence *n* ≤ 38,181	Bivariate genetic modelling Current smoking-coffee intake: G r = 0.47, E r = 0.30 Current smoking-caffeine use: G r = 0.44, E r = 0.00 MR: NS LD score regression Smoking heaviness- coffee intake: r = 0.44 Smoking initiation-coffee intake: r = 0.28 Smoking persistence-coffee intake: r = 0.25	Genetic factors explain most of the association between smoking and caffeine consumption. Quitting smoking may be more difficult for heavy caffeine consumers, given their genetic susceptibility.	Underpowered Pleiotropy
Taylor et al., 2017 [47]	Prostate cancer (PC) risk and progression	2-SNPs *AHR, CYP1A2*	Individual-level data Two-sample MR Regression analyses + meta-analysis Practical consortium (*n* = 46,687) 4 studies GS-coffee GS-tea GS-(tea + coffee) 23 studies GS-PC GS-PC stage GS-PC grade GS-mortality	Significant GS-coffee, GS-tea and GS-(tea + coffee) GS-PC grade (*p* = 0.02)	No clear evidence supporting a causal relationship between coffee and outcomes	Between-study heterogeneity in case definition Imprecise IV Pleiotropy Underpowered
Ware et al., 2017 [48]	Smoking heaviness, cigs/day	8-SNP GS *AHR, CYP1A2, GCKR, MLXIPL, POR, EFCAB5, BDNF, ABCG2* 6-SNP GS *AHR, CYP1A2, GCKR, MLXIPL, POR, EFCAB5* 2-SNP GS *AHR, CYP1A2*	2-sample MR Summary-level data IVW, WME CCGC TAG GWAS Cotinine levels (*n* = 4548) [in vitro experiments] Individual-level data (replication, *n* = 8072 smokers who drink coffee) IVW, WME	Each cup of coffee/day lead to a decrease in 1.5 (8 SNPs), 1.7 (6 SNPs) or 2.0 (2 SNPs) cigs/day. Coffee did not influence cotinine levels. Coffee did not influence cigs/day in replication sample.	Coffee intake is unlikely to have a major causal impact on cigarette smoking	Pleiotropy Underpowered replication Underpowered IV
Bjorngaard et al., 2017 [49]	Coffee intake (cups/day, sensitivity analysis: Any vs. none) Tea intake (cups/day, sensitivity analysis: Any vs. none) Smoking status (never, former, current) Smoking heaviness (cigs/day)	1-SNP (*CHRNA3*) for smoking heaviness 2-SNPs (*AHR, CYP1A2*) for coffee intake GS	Individual-level data Bidirectional MR Regression analyses + meta-analysis UK biobank (*n* ≤ 114,029) HUNT (*n* ≤ 56,664) CGPS (*n* ≤ 78,650) coffee or tea drinkers only	Observational Former & current smoking associated with higher coffee consumption (not tea) vs. never smokers. Among smokers: Each cig/day increased coffee and tea intake; stronger for coffee MR SMK-SNP associated with coffee intake in current or ever smokers only Coffee-SNP not associated with smoking behavior	Higher cigarette consumption causally increases coffee intake.	Underpowered to rule out causal coffee → smoking association. UK Biobank non-representative sample Collider bias: (i) if selection into the sample is related to both coffee and smoking (ii) via smoking stratification Phenotype measurement error
Larsson et al., 2017 [50]	Alzheimer’s Disease (AD)	5-SNP GS *AHR, CYP1A2, MLXIPL, POR, EFCAB5* (coffee and 23 other exposures tested)	Summary-level data 2-sample MR IVW, WME, MR Egger CCGC International Genomics of Alzheimer’s Project (*n* = 17,009 cases, 37,154 controls)	Suggestive association between coffee GS and increased risk of AD (*p* = 0.01)	Suggestive causal relationship between coffee and AD risk, but in opposite direction to that expected based on observational studies.	None.
Verweij et al., 2018 [51]	Causal associations between nicotine, alcohol, caffeine, and cannabis use	Polygenic scores (*p* < 5 × 10^−8^ or *p* < 1 × 10^−5^) for each exposure	Summary-level data two-sample bidirectional MR IVW, Wald ratio Multiple published GWAS	Smoking cigs/day—caffeine use (*p* = 0.01) Alcohol use: Smoking initiation (*p* = 0.03)	Little evidence for causal relationships between nicotine, alcohol, caffeine, and cannabis use, but may suggest a common liability model (shared genetics)	Imprecise IV GWAS sample overlap (bias to null)
Ong et al., 2017 [52]	Epithelial ovarian cancer	4-SNP GS (coffee IV) *ABCG2, AHR, CYP1A2, POR* 2-SNP GS (caffeine IV) *AHR, CYP1A2*	Summary-level data Two-sample MR Wald-type ratio estimator CCGC Ovarian Cancer Association Consortium (*n* = 44,062, 20,683 cases)	NS	No evidence supporting a causal relationship between coffee/caffeine and outcome	MR Assumption 3 not confirmed Not generalizable to non-European populations. Underpowered or imprecise IV Cannot rule out non-linear effects of coffee/caffeine on cancer
Larsson et al., 2018 [53]	Gout	5-SNPs *AHR, CYP1A2, MLXIPL, POR, EFCAB5*	Summary-level data 2-sample MR IVW, WME, MR Egger CCGS Serum Uric acid GWAS (*n* = 110,347) Gout GWAS (2115 cases and 67,259 controls).	*CYP1A2* and *MLXIPL* SNPs inversely associated with uric acid Combined MR: significant inverse relationship (*p* = 7.9 × 10^−6^) All but *AHR* SNP associated with lower gout risk. Combined MR: significant inverse relationship (*p* = 0.005)	Supports causal inverse association between coffee intake and risk of gout.	None
Treur et al., 2018 [54]	Sleep behaviors (sleep duration, chronotype and insomnia complaints)	IV threshold *p* < 5 × 10^−8^ 4 SNPs (*POR, AHR, CYP1A2, MXLIPL*) *p* < 5 × 10^−5^ 4 SNPs plus 23 SNPs	Summary-level data Two-sample bidirectional MR IVW, LD score regression CCGC Caffeine metabolite GWAS Sleep GWAS	MR: NS LD score regression: NS	No evidence for causal relationship between habitual coffee intake and sleep behaviors.	Underpowerd LD score regression using caffeine metabolite GWAS Phenotype measurement error
Noyce et al., 2018 [55]	Parkinson’s Disease (PD)	Morning person primary exposure (15 SNPs) coffee secondary exposure (4-SNPs, *AHR, BDNF, POR, CYP1A2*)	Summary-level data Two-sample MR IVW CCGC Morning person GWAS (*n* = 89,283) PD GWAS (13,708 cases, 95,282 controls)	Morning person MR: *p* = 0.01 Coffee MR: NS	Along with published RCT results, findings suggest that caffeine may neither prevent PD occurring nor be of benefit in those with the condition.	Use of summary-level data does not allow adjustment for potential confounding factors.
Zhou et al. 2018 [56]	Cognitive function composite global cognition and memory scores	2-SNPs *AHR, CYP1A2* Other SNPs (secondary analysis)	Individual-level data *n* = 415,530 (300,760 coffee drinkers) from 10 meta-analyzed European ancestry cohorts. Genetic analysis performed under different levels of habitual coffee intake (1–4 and ≥4 cups/day. Negative control: Non-coffee drinkers.	Observational: No overall association between coffee intake and global cognition and memory. SNP-outcome: NS	Study provides no evidence to support beneficial or adverse long-term effects of coffee intake on global cognition or memory.	Pleiotropy. Caution when interpreting coffee IV
Lee, 2018 [57]	Osteoarthritis	4 SNPs, *POR, CYP1A2, NRCAM, NCALD*	Summary-level data Two-sample MR IVW, WME, MR-Egger regression CCGC + Amin et al. 2012 (*n* = 18,176) Osteoarthritis GWAS (7410 cases, 11,009 controls)	IVW: *p =* 0.03 WME: *p* = 0.05 MR Egger: NS (however, no pleiotropy was evident)	Results suggest that coffee consumption is causally associated with an increased risk of osteoarthritis.	Underpowered or imprecise IV Results limited to populations of European ancestry and limited to osteoarthritis in the knee and hip

AD—Alzheimer’s disease; BMI—body mass index; CCGC—Coffee and Caffeine Genetics Consortium; DBP—diastolic blood pressure; DIAGRAM—Diabetes Genetics Replication and Meta-analysis; GS—genetic (SNP) score; HDL—high-density lipoprotein; IHD—ischaemic heard disease, IS—ischaemic stroke, IVD—ischaemic vascular disease, IVW—inverse-variance weighted meta-analysis, NS—non-significant; PC—prostate cancer; PD—Parkinson’s Disease; SBP—systolic blood pressure; T2D—type 2 diabetes; TC—total cholesterol; TGs—triglycerides; WC—waist circumference; WME—weighted median estimate.
